# On the Prognosis and Treatment of Proliferous Inflammation of the Middle Ear

**Published:** 1894-09

**Authors:** Lucien Howe

**Affiliations:** M. R. C. S., Eng.; 183 Delaware Avenue


Buffalo Medical and Surgical Journal

VOL. XXXIV. SEPTEMBER, 1894. No. 2.

### Original Communications.

### ON THE PROGNOSIS AND TREATMENT OF PROLIFEROUS INFLAMMATION OF THE MIDDLE EAR.

BY LUCIEN HOWE, M. R. C. S., Eng.

AS THE nomenclature of diseases of the middle ear is not as clear as it should be, a word of introduction to this paper is necessary in order to point out the class of cases here referred to. The non-suppurative inflammations of the middle ear are undoubtedly due, to a great extent, to that pathological condition which we call catarrhal! But the word catarrhal is misleading, both to the laity and to the profession, giving an erroneous idea as to the cause upon which the disease depends and the treatment indicated. It is better, therefore, to use the word proliferous, as the translation of the German *Wucherung*, as Roosa has done, to indicate that very large class of cases which begin as a simple inflammation of the mucous membrane of the middle ear, though sometimes without any such condition distinguishable at an early stage, but where there results a thickening of that membrane. This inflammation usually extends from the pharynx to the Eustachian tube and into the middle ear, producing changes in the membrana tympani, especially so impeding the movement of the ossicles as to produce imperfect hearing, and finally leading to a series of pathological changes which result in more or less complete deafness.

It is not my intention to give any description of the clinical history or symptoms of these cases. Indeed, some patients suffering from the proliferous form have no pain nor anything else to attract attention, unless it be a singing, roaring or buzzing sound—the tinnitus. This, in fact, is often the only symptom which gives any discomfort, but, when the hearing is tested, the patient finds that one ear is partly or entirely deaf, and that a similar difficulty has perhaps begun to show itself on the other side.

It is unquestionably the fact that the most important class of cases of impaired hearing are those due to this form of chronic inflammation of the middle ear. The disease in any variety is important because of its frequency, its insidious course and its tendency to result in deafness more or less complete. As to its frequency, statistics vary somewhat, but it is safe to say that considerably more than 45 per cent. of all the cases presenting themselves for treatment are those of chronic proliferous inflammation of the middle ear.

In forming an opinion as to the prognosis of any special case, of course, the anatomical condition is of first importance. If the drum head is but slightly retracted, if inflation of the middle ear shows that the ossicles are easily movable, especially if the tube be open, and the hearing but slightly impaired, of course, the prognosis is favorable. On the other hand, as these factors change, the prognosis naturally must also be varied. To touch upon the importance of each of these points would be to elaborate the pathology of this whole class of diseases. In so short a paper as this, a description of the changes in the middle ear itself must be left out of account, these being different in each individual, and we must content ourselves with a few general but exceedingly practical considerations which influence the prognosis in these cases. Among these I may mention, foremost, the age of the patient.

When any such inflammation of the middle ear occurs in early life, it is almost invariably amenable to treatment. Then the prognosis is excellent. Ordinarily, too, it is so in the more or less acute stage, and many of the patients who apply with the hearing distance reduced to half, quarter or even a tenth of the normal, can be made to hear, ultimately, perfectly well, as measured by the most exact tests. Even a little later in life the prognosis is good, when the disease is recognized in an early stage and where efforts are pursued in a thorough and intelligent manner during a considerable time.

In the second place, the question of hereditary tendency is also quite an important element in estimating the prospects for the patient. We have long ago learned that this inherited tendency to deafness is not any mysterious curse or ban placed upon certain families. But, as in a large proportion of all cases of inflammation of the middle ear, the difficulty commences in a Eustachian tube smaller than normal or not as straight as it should be, so do we find, in certain families, the Eustachian tube not only smaller than normal but also more liable to collapse, or, by its slightly irregular form, also more liable to the retention of any mucus which may accumulate in it. As such tubes have a greater tendency to become closed than others, members of these families are more liable to deafness at an early age, and to have it in a more obstinate form, than those where this peculiarity does not exist. The fact is, as I repeat, that there is, with many, an inherited tendency to inflammation of the middle ear, due to this or similar physical causes, and this tendency forms an important element in estimating the prospects of the patient for the cure or arrest of the trouble with which he is afflicted.

Various other elements must be taken into account in estimating the prospects of the patient. The condition of the health is not to be forgotten, for anemic individuals and those exhausted by illness have less recuperative power, of course, than the strong and healthy.

Finally, a long experience has taught me that the mental caliber and strength of mind is by no means to be left out of account. The patient who begins his treatment with impulsive and spasmodic energy is one to be easily discouraged, to fold his hands in an aimless way and give up the battle which he must make against the disease. With such a person, the prospects are unfavorable. On the other hand, with a quiet determination and strength of will sufficient for patient persistence in the treatment, and with the determination to overcome the obstacles by perseverance through weeks and months of care with little or no apparent gain,—for such a one the prospects are infinitely better.

In this connection, I am reminded of a case which came to me, at least twelve or fifteen years ago, of an unusually intelligent woman, prominent as an amateur musician, whose husband is one of the leading lawyers of his city. There were many points of discouragement in the case, and my prognosis was by no means favorable, but I insisted upon the necessity of such care as could be given at home, not for weeks but for months and even years. I have often listened, since then, to her admirable execution as a pianist, and noticed in that way, and by conversation with her, how well her hearing has been retained. Several years after, I spoke of the matter to her husband. "Doctor," he said, "I do not think I have omitted the use of that inflator more than once or twice since those instructions were given." To his persistence and her patience that result can be credited. A less intelligent patient with the same difficulty would have been deaf in a comparatively short time.

In summing up all of the points of the prognosis to be given to the patient, it is well, I think, to acknowledge to ourselves how much still remains to be learned, and to acknowledge frankly to the patient how much must not be expected. As I have already indicated, after the disease has once obtained a good foothold, cure is practically out of the question. The sooner this is said directly and promptly, the better it is for the comfort of the patient and the credit of the practitioner. Of course, it is a shock, often a severe one, especially so when the comparative youth of the patient, or special circumstances or conditions, make the loss of hearing an unusually great deprivation. But in the present state of science we are practically unable to effect anything like perfect cure in the cases far advanced ; great improvement is also rare, and this question being the uppermost in the patient's mind, should be answered immediately, directly and explicitly.

The prospects of moderate improvement, however, can be stated more encouragingly. In many cases the circumstances may be such as to warrant considerable expectation in that respect, but I find that the oldest practitioners, and those with the largest experience, are also those the least apt to commit themselves upon this point. They simply say, all that we know what to do, shall be done ; but the condition of the ear and the symptoms must be unusually promising to enable one to hold out hopes of very great improvement after such a chronic disease of the middle ear has become well established and is advancing.

But if the disease cannot be cured, if it cannot even be improved, the patient asks finally whether it can be arrested. It is in this that the great value of the treatment lies. I know that various opinions exist as to what may be expected even in this phase of the matter. I have myself had cases, and also seen others in the care of those whose experience is quite as great as mine, go steadily from bad to worse in spite of everything that could be done. The advances in otology have been sufficient to enable it to be said without the least fear of contradiction, that we do know how to arrest the progress of such an inflammation of the middle ear in the great majority of cases, and for the remainder, where progress from bad to worse still goes on, that progress is often unquestionably made much slower by proper treatment.

It is unnecessary here to array a long list of figures in support of this proposition, as could easily be done. I think no one fails to notice the great difference in this respect in the ultimate results between patients seen at a private office and at a public institution. In my own practice, the poorest patient treated at the infirmary has his case recorded with as much exactness as any one of those at my office. When a private patient is told what to do in order to hold in check the progress of this middle ear inflammation, he usually goes home to consider the matter, and returns after a while for such care as is necessary, or for such directions as he is ready to follow out at home, either alone or under the supervision of his family physician. And usually he carries out these directions with a certain degree of thoroughness. On the other hand, the infirmary patient has, as a rule, only one question to ask. He says, first of all, Can you cure me? and if his case is one of these now under consideration, and is far advanced, the answer is briefly and promptly, No. He is always disappointed—usually disgusted. He says he has no time to spare from his work in an attempt to ward off what may not come. He is ready to "risk it," as he says, and proceeds to do so. But that patient is apt to return. It may be in six months, or a year, or six years, but whenever he does, the condition of the ears then can be compared with the record made at the time of his first visit. There is almost invariable loss, and the amount lost is certainly greater by far than with those who struggle to retain what hearing they have. Of course, one or two dozen cases of either kind prove nothing, but when it is possible to put together over seven hundred of the one kind, and offset those with just as many taken just in the same way, of the other kind, we do find, beyond a shadow of doubt, and with a certainty which is far removed from the errors of small statistical tables, that the average of those who have been systematically treated, or who have themselves followed out such a course, is infinitely better than the average of the careless and negligent, who are thus ready to "take the risk."

We come now to consider the best means of treating a typical case of chronic proliferous inflammation of the middle ear. As the difficulty often begins in the Eustachian tube, and with an inflammation of the pharynx, it is convenient in considering this question to commence with that.

The first and most important factor in this connection is cleanliness. This cleanliness may be that which is obtained by simple washing of the mucous membrane and the lower portion of the Eustachian tube, or it may be the chemical cleanliness obtained by antiseptics. For the first, one of the simplest and most efficacious methods is to inhale water through the nostrils. Pure water is apt to produce a stinging of the mucous membrane of the nostril, but if the specific gravity is changed by adding to a glassful of water a teaspoonful of salt, it will be possible then to inhale the solution in such a way as to wash out the upper and posterior nares, and thus partially to cleanse the lower portion of the Eustachian tube. Such an attempt is, however, to a certain extent, rather unsatisfactory, for the reason that the water which passes into the nostril simply runs down over the Eustachian tube, very much as the water from a roof runs over the eaves, not reaching up underneath them. In order to obviate this difficulty, it is well to syringe out the upper pharynx by means of a posterior nasal syringe, or so-called catarrhal syringe, using a solution of bicarbonate of soda or other weak alkaline mixture. Most of the syringes have a bulbous extremity which is perforated, and through which the water distributes itself over the whole membrane. This is not as efficient or satisfactory as is a single stream, and, for that reason, I am accustomed to have my patients buy the ordinary syringe, and then, cutting off the bulbous extremity and smoothing it well, warm the end and give the tip a curve almost at right angles. This end can then be introduced without difficulty under one side of the palate and, by turning the opening a little outwards, the stream can be forced not only against the lower opening of the Eustachian tube, but practically up into it. By varying this solution we can obtain not only the cleanliness which washes away all the existing secretion, but also the chemical cleanliness which, as far as may be possible under such circumstances, disinfects the secretion already there.

The value of the nasal douche in treating disease of the middle ear is a disputed question. Roosa and one or two others are strongly opposed to its use in any form, insisting that injury has been caused by it and that the results at best are unsatisfactory, while, on the other hand, Burnett and some of the most excellent German authorities contend that if used cautiously and with little or no force, it is possible in this manner to wash out the posterior nares easily and thoroughly. In general, therefore, we may conclude that it is to be used with some caution and only under the direction of a physician.

After having once thoroughly cleansed the lower portion of the Eustachian tube and the membrane in that vicinity of the accumulated mucus and pus which may be adherent to the membrane, it is then in a condition to receive with advantage any astringent or weak caustic applications that may be made to it. One of the most reliable agents for this purpose is a solution of nitrate of silver, of one, two or even eight per cent. For twenty years I have watched with interest the fate of other remedies which have been brought forward to take the place of nitrate of silver, have seen them come into fashion and disappear, leaving that as the substance to which we must pin our faith in the treatment of the mucous membrane in this class of cases. It requires, however, no small amount of dexterity to make the application properly. On that account it is often neglected. Sponge probangs are of little or no use in this respect, although they are often recommended and sometimes employed. A camel's hair brush may be bent at a proper angle and attached to a good handle—as a penholder—and then, if the tongue is forcibly depressed, the brush can be first touched to the posterior wall of the pharynx, with the bristles bent downwards, and then, when the patient "gags," the bristles are quickly turned upward and the brush pushed upward and outward, while the handle is forcibly depressed against the opposite angle of the mouth.

Under this heading, also, it is well to mention the various applications, many of undoubted merit, which are frequently used in the form of sprays. Just at present menthol spray is the favorite, five to ten grains to the ounce of alboline making a pleasant stimulant and one which, in many cases, is of undoubted benefit. I think, however, that too great stress is laid upon the advantage of these, where the time and energy of the patient could be better expended in caring more directly for the cleanliness of the tube, or making astringent applications to it, or even to its dilatation, as will be mentioned presently, by means of the Eustachian inflator.

Having succeeded in cleansing the pharynx and the lower opening of the tube, and having used such stimulating or astringent applications as are necessary, the next step is to open—"inflate"—the tube, which is almost invariably partly or entirely closed. One of the oldest and simplest methods of accomplishing this is the Valsalva's inflation. This consists, as is well known, in filling the lungs well with air, then holding the nostrils closed and closing the mouth also ; an expiration drives the air upwards, which, being unable to escape through the nose or mouth, passes into the Eustachian tube. This inflation of the ears has been made by nearly every person when using the handkerchief, when by chance the nostrils and mouth have been closed at the same instant. The air was pressed upwards from the lungs, and the individual has felt a peculiar clicking in one or both ears. The object of the Valsalva's inflation is only to accomplish this. Simple as the procedure appears, it is not always done on the first trial, but with a little tact and patience one easily succeeds, and having been done once the patient repeats it with little or no effort. If the tube has closed firmly and it is impossible to press air through it, the experiment can be repeated four or five times a day, especially if the disease then exists in a more or less pronounced catarrhal form. The method is so simple, that the patient can be instructed to follow it with little or no hesitation or fear of doing harm.

Next to the Valsalva's inflation of the middle ear, the method advised by Politzer is the simplest. At his suggestion, as is well known, the air is forced from a gutta-percha bag into one nostril, through a plug which fits that nostril tightly, while the opposite nostril and mouth are closed. The patient is instructed to swallow at the same instant that pressure is made on the rubber bulb. In doing this the patient causes the soft palate to be thrown horizontally backwards when he swallows, and if at that instant the air is forced from the bag into the nostril, it must enter the Eustachian tube, as it cannot escape by the other nostril or downward into the mouth. With a little instruction most patients are able to carry out this portion of the treatment themselves, and, for my part, I am accustomed to have them, or some member of the family, take practically entire charge of this part of the detail. I know that other practitioners and writers consider that this involves risk. Many advise the use of the Politzer-bag only by the trained hand of a skilful practitioner, and I also find a few otherwise intelligent persons who are incapable of carrying out this part of their treatment. But I must confess I do not feel warranted in advising office patients to add to their expense and annoyance by many visits, when, by proper instruction, this part of the treatment can be followed, at least in part, so much more conveniently at home. For many also who find it out of the question to come for any treatment, especially those who live at a considerable distance from the city, it is a great advantage to enable them to assist themselves by the intelligent use of the Politzer-bag.

There are other methods of inflating the Eustachian tube, such as have been recommended by Gruber and others, but it is unnecessary, in so short a paper, to dwell upon these modifications at any length. They all depend upon the principle of causing the soft palate to shut off the upper portion of the pharynx from the lower, while air is forced into the nostril, and thence into the Eustachian tube and middle ear. Nor is it necessary, in this connection, even to mention those procedures by which the air from the middle ear is drawn out, the object of this paper being to present the salient points of treatment only of the disease under consideration.

By far the most efficacious method of treating this, and, I may add, all allied diseases of the middle ear, is by means of the Eustachian catheter. Since it was first shown how that could be passed through the nostril into the tube, and what favorable effects could be obtained by this simple instrument, it has been held in the highest esteem by those whose experience is largest. It must be admitted that the introduction of the Eustachian catheter requires some experience and care. I have noticed, when instructing students, that very few are able at first to introduce the point of the catheter into the Eustachian tube, no matter how explicit are the directions given to them or how carefully they have studied the subject in text-books. Moreover, it is undeniably the fact that unfortunate results have occurred from its careless use. In some well-authenticated cases the end of the catheter has been pushed into the cellular tissue of the pharynx, and the surgeon has inflated the whole areolar tissue in that vicinity instead of the tube, producing pain, annoyance and, in one or two cases, more serious results. But I feel justified in saying that, with a little experience, it is one of the safest manipulations in surgery. When this catheter is properly passed through the nostril into the Eustachian tube, the simple inflation by air alone is one of the most efficacious means of dilating the closed tube. In the class of cases under consideration, it is necessary to repeat this introduction of the catheter day after day, and sometimes week after week, though in a short time a thorough inflation in this way will be sufficient if made every three or four days or, perhaps, once in a few weeks. The apparent results are slow, but are those upon which we can rely with more confidence than upon any other method of treatment.

In passing, I may mention that in the acute form of aural catarrh there is nothing which compares with the Eustachian catheter in the satisfactory results which it gives. I think nearly every one can corroborate what Roosa says of the delight occasionally exhibited by patients when, after having been practically shut off from the world by their deafness, air is pushed through the catheter into the tube. Suddenly the face brightens; they exclaim: "Doctor, I am well. I hear again." Unfortunately this result is never found after the disease has advanced to a chronic stage, or passed into that now under consideration, but with persistent effort, for a considerable time, we find that this inflation yields better results than any other method.

The Eustachian catheter may also be used for the introduction of steam or medicated vapors into the tube, and many of these are unquestionably of some benefit, especially in the class of cases which are more or less of a pronounced catarrhal form. To these it is worth while, in most instances, to give a fair trial. On the other hand, I cannot agree with those who go so far as to advocate or prescribe introduction of bougies, through such a catheter, into the Eustachian tube or the middle ear, or any of the forcible efforts to dilate the tube. They are of questionable benefit, and not without some danger; and above all, we must be careful that in attempting to improve, no risk is taken of making the patient worse.

Finally, it is natural to ask ourselves if something cannot be accomplished, with old and desperate cases, by operation or by other methods more active than the simple means to which I have referred. This is a field which has been worked earnestly and conscientiously for a long while, and it must be confessed that until recently the results have been by no means encouraging. The operation of myringotomy unquestionably does very frequently lessen the annoying roaring in the ears, and, like others, I have passed through the stage when, encouraged by these favorable clinical reports, I have several times divided freely the membrane in certain cases of this class, but usually it healed promptly and the noises returned. Nor have I obtained much better results in five cases in which the opening was burned through with chromic acid, or with the galvano-cautery—a procedure requiring considerable care, of course. It is undoubtedly the fact, though, that if we could make a smooth, clean opening in the drum-head, and keep that opening from healing, it would be possible to medicate the middle ear more thoroughly and intelligently. For this so eminent an authority as Burnett, with others, still advocate the introduction of an eyelet through an incision made in the drum-head, in order that the aperture may be retained, but it is a procedure difficult to carry out, and the results thus far have not been satisfactory, plausible and attractive as it may seem in theory.

It has long been known that where a considerable opening existed in the drum of the ear, not only did the person hear perfectly well for practical purposes, but it was noticed that in many cases where suppuration occurred, and such an opening did remain in one ear, the amount of hearing was greater than that remaining in the other ear, where practically the same process had gone on, except as regards the opening. This surgical procedure of nature had long ago taught aural surgeons that such an opening is not only desirable, but a necessity to hearing in certain cases, and it is not too much to expect that they will soon be able to maintain such an opening in the membrane as to treat the middle ear with astringents and other proper solutions as readily as such applications are made to the inner surfaces of the eyelids.

Another procedure which gives promise of most satisfactory results is the so-called mobilization of the bones—especially the stapes. An article like this can make only the merest reference to this step in advance. In another place I hope to discuss this method of treatment more in detail. The fact is evident that immobility of the bones of the ear is a very important factor in the production of imperfect hearing, and the problem before us is very similar to that which confronts the general surgeon in the case of a stiff-jointed knee or elbow. In that case, he institutes forcible motion and the bones move upon each other with more or less freedom afterwards. In the ear the problem is to move these adherent bones one upon the other, and especially to lessen the adhesions which in most instances the stapes has had formed about it. The brilliant results obtained in some cases and the many instances of decided improvement in others, give us strong reason to hope that this operation is one destined to bring relief to many in the future. Even a small experience with it justifies this conclusion. As for the entire removal of the stapes, as advocated by Jack, this operation, it must be admitted, is still to be held as under trial, and considered as applicable to only a small class of cases. In general, I think nearly every practitioner of experience will agree with me that when he compares his results during the last four or five years with those in the earlier years of his practice, he sees that the advances made in operations on the middle ear have done much to warrant encouragement in a class of cases which before we saw go from bad to worse, while we confessed with shame our ignorance and inability to give the relief so much desired.

183 DELAWARE AVENUE.

